# Integrated Single-Cell RNA-Sequencing Analysis of Gastric Cancer Identifies FABP1 as a Novel Prognostic Biomarker

**DOI:** 10.1155/2022/4761403

**Published:** 2022-06-28

**Authors:** Fan Yang, Lianfang Gan, Junhua Pan, Yaying Chen, Hong Zhang, Ling Huang

**Affiliations:** ^1^Hainan Province Key Laboratory for Drug Preclinical Study of Pharmacology and Toxicology Research, Hainan Medical University, Haikou 571199, China; ^2^College of Traditional Chinese Medicine, Hainan Medical University, Haikou 571199, China; ^3^Research Center for Drug Safety Evaluation of Hainan Province, Haikou 571199, China; ^4^State Key Laboratory of Trauma, Burns and Combined Injury, Department of Wound Infection and Drug, Daping Hospital, Army Medical University, Chongqing 400042, China

## Abstract

Gastric cancer (GC) is usually diagnosed in an advanced stage at the first visit due to the atypical clinical symptoms. The low surgical resection rate and chemotherapy sensitivity result in dismal survival. Therefore, it is urgent to develop novel biomarkers with high sensitivity and specificity to accurately assess the prognosis of GC patients. In the present study, 3385 differentially expressed genes (DEGs) were obtained from the single-cell RNA sequencing data of GC specimens. Using the unsupervised dimensionality reduction, we further found 3 subsets of cells including gastric cells, plasmacytoid dendritic cells, and memory T cells. Based on the cell clustering, we explored the key regulatory genes for GC progression by pseudo-time analysis and functional enrichment analysis. According to the results, the significant differentially expressed fatty acid-binding protein 1 (FABP1) verified by pseudo-time analysis was identified as the hub gene of GC progression. FABP1 was shown to be closely related to the long-term survival and the age at diagnosis of patients with GC in analysis based on the TCGA (The Cancer Genome Atlas) database. To further verify the role of FABP1 in GC, we performed immunohistochemical (IHC) analysis using the GC tissue microarray and found that the expression level of FABP1 was higher in GC tissues than in the adjacent tissues. Moreover, GC patients with higher expression of FABP1 had a worse clinical outcome. In summary, our study revealed that FABP1 is a potential effective biomarker for the prognosis of GC, and high expression of FABP1 predicts unsatisfactory survival.

## 1. Introduction

Gastric cancer (GC) is one of the most common gastrointestinal cancers in the world, with the second-highest mortality rate [1]. Global GC incidence varies widely, with the highest incidence in East Asia [1]. The current treatment strategies for GC are mainly surgery, chemotherapy, radio-chemotherapy, and targeted therapy. However, chemotherapy resistance and high postoperative cancer recurrence rate usually lead to cancer-related death [2, 3]. Therefore, it is critical to study the molecular mechanisms of gastric cancer progress and to identify novel biomarkers for early gastric cancer.

Recently, single-cell RNA sequencing (scRNA-seq) technology has made significant progress and is widely used in the study of various cancers [4]. This approach is crucial for the discovery, identification, and validation of new biomarkers. Individualized clinical treatment will also be the goal of scRNA-seq technology [5].

Fatty acid-binding proteins (FABPs) are a group of highly conserved cytoplasmic proteins with a low molecular weight [6]. As lipid chaperones, FABPs mainly solubilize fatty acids, which are involved in intracellular metabolism and other signaling processes [7]. At present, more than nine kinds of FABPs have been identified. The human FABP1 gene was first found in the liver, located on chromosome 2, encoding a protein of 127 amino acids (aa). FABP1 protein has a classical *β* structural fold and two short alpha-helices [8–10]. FABP1 has multiple functions, such as a protective agent against oxidative stress, and is involved in the regulation of adipogenesis and lipid metabolism [11]. In addition, FABP1 is associated with various diseases such as liver fibrosis, nonalcoholic steatohepatitis, acute kidney injury, renal ischemia/reperfusion injury, type I diabetes, and type II diabetes [12]. Studies have shown that the expression level of FABP1 is closely related to the occurrence and progression of various tumors. FABP1 is found highly expressed in Barrett's esophagus [13]. The expression of FABP1 is also positively correlated with the incidence of pancreatic cancer, especially the diabetes-related pancreatic cancer [14]. FABP1 has also been found to be downregulated in cancers. Low expression of FABP1 was found in the early-stage colorectal cancer and 93% of microsatellite unstable colorectal cancers [15, 16].

In this study, we analyzed the single-cell sequencing data of gastric cancer and found that FABP1 was one of the most significant differentially expressed genes (DEGs) in GC tissues. FABP1 was identified as the hub gene in GC progression. The TCGA analysis also showed that FABP1 was closely related to the prognosis of GC patients. The IHC analysis based on GC tissue microarray further verified that FABP1 was highly expressed in GC tissues, confirming that GC patients with higher expression of FABP1 have a lower long-term survival rate. Therefore, we proposed that FABP1 is a promising biomarker for GC.

## 2. Methods

### 2.1. Data Acquisition and Preprocessing

**Figure 1:**
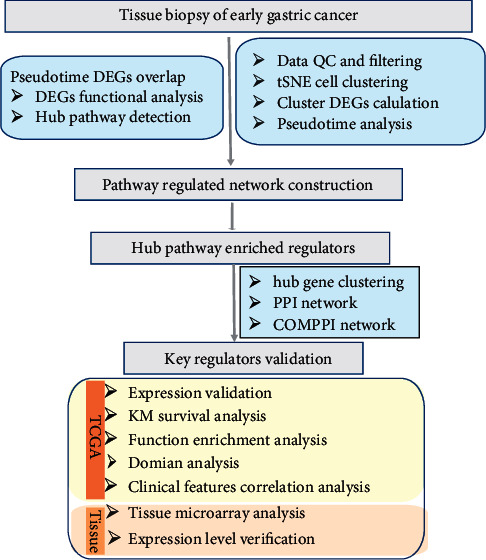
The study flow chart.

After searching through the Gene Expression Omnibus (GEO, http://ncbi.nlm.nih.gov/geo/) database [17], we downloaded the reads per kilo base per million mapped reads (RPKM) scRNA-seq data from the GSE134520 [18]. The data were constructed by thirteen gastric antral mucosa biopsies with the pathologic diagnosis including nonatrophic gastritis (NAG), chronic atrophic gastritis (CAG), intestinal metaplasia [17], or early gastric cancer (EGC). These data also match the platform annotation of GPL20795 HiSeq X Ten (*Homo sapiens*) [18]. The study flow chart is shown in Figure 1.

During data preprocessing, we read the original expression values by the Seurat function, and the number of genes and the Unique Molecular Identifiers (UMIs), representing the non-normalized expressional values within a cell, were automatically calculated [19, 20]. The sum of the percentages for mitochondrial was calculated with the criterion of filtration of 5%. Here, cells with expressed genes <100 and genes expressed in <3 cells were removed from the dataset. The LogNormalize algorithm was used to normalize the original data, and the FindVariableGenes algorithm was used to find the variable features [19, 20].

In addition, based on the orthogonal transformation algorithm, the principal component analysis (PCA) analysis was applied to the dimension reduction process of scRNA-seq data to highlight data features in lower dimensions [21]. After selecting the key components, the important dedicator contributing to data differentiation, the tSNE method was selected to detect the cell subtypes with the data resolution of 1.0(3, 4). For the patterns of the gene expression matrix of a cell corresponding to different cell subtypes, we selected the SingleR and scCATCH methods to identify the cell subtypes [19, 20, 22].

### 2.2. Differential Gene Analysis

The FindAllMarkers function is a frequently used method to detect the differentially expressed genes (DEGs), based on the Wilcox analysis, with the criterion of log |fold changes (FC)| over 0.25 and *P* < 0.05 after FDR correction [19].

### 2.3. Analysis of Biological Functions of Differential Genes

Using the clusterProfiler package and MetaScape database (http://metascape.org/gp/index.html#/main/step1), we further analyzed the Gene Ontology (GO) [23] and Kyoto Encyclopedia of Genes and Genomes (KEGG) pathway enrichment, respectively [24]. The MetaScape database is an international authoritative functional database of gene annotation, visualization, and integrated exploration, facilitating the integration of genetic pathways and functional enrichment. A P-value <0.05 was considered statistically significant in our GO function and KEGG pathway analysis.

### 2.4. Pseudo-Time Analysis and Pseudo-Time DEG Analysis

Many of the cellular states of the various cell fate processes are not perfectly synchronized, some cells are at the beginning of a particular process, while others are already at the completion of that process, which is also known as “asynchronous.” Based on the Monocle reverse embedding graph algorithm, we quantified the transformation under different cell states and the transcription state of the corresponding gene set and form a trajectory by sorting these cells according to this transcriptional process, thus tracking the process change function that accompanies the trajectory, called pseudo-time analysis. Pseudo-time is an abstract unit of differentiation, which is only a distance from the cell to the start of the trajectory, measured along the shortest path. By estimating SizeFactors algorithm, we evaluated the gene expression range of each cell and used it for subsequent normalization and calculation of gene variance. Differential GeneTest algorithm is used to calculate the differential core regulatory genes in pseudo-time analysis, and B-H correction *P* value <0.01 is considered to be pseudo-time DEGs.

### 2.5. Analysis of Protein Interaction Networks Based on DEGs

After deriving the DEGs of the hub pathway, we performed the core expression analysis in a volcano map based on the average Log |FC| and Negative Log10 (adjusted *P* value). Subsequently, the hub tagged target proteins were calculated based on the COMPPI database (http://comppi.linkgroup.hu/) [25]. The COMMPI platform was used to integrate the subcellular localizations, protein-protein interactions, and scores of localizations and interactions, with the locations of cytosol, mitochondrion, nucleus, extracellular secretory pathway, and membrane.

### 2.6. Functional Enrichment and Prognosis Analysis of Hub Genes

We used the ToppGene database (https://toppgene.cchmc.org/) to annotate and visualize the hub marker's functional enrichment based on Gene Ontology [23] and pathways [26]. The terms with *P* < 0.05 were significantly enriched. To gain further insight into the hub gene expression and its association with prognosis, we applied a friendly online web tool Wanderer (http://maplab.imppc.org/wanderer/) to identify the important clinical features correlated with transcriptional expression. Additionally, the SurvExpress (http://bioinformatica.mty.itesm.mx:8080/Biomatec/SurvivaX.jsp) online database was applied to validate the prognostic relationship among the FABP1 expressions [23]. Besides, the statistical difference was considered with the *P* value <0.05.

### 2.7. Immunohistochemical Analysis and Statistical Analysis

Gastric cancer tissue microarray (TMA, HStmA180Su19) was obtained from Shanghai Outdo Biotech, including 94 cases of gastric cancer tissues and 86 cases of adjacent tissues; with complete case data and follow-up information, more detailed sample information and clinical features of colorectal cancer are shown in Table 1. The IHC assays and IHC scores were performed with a previously described protocol [27], and the antibodies against FABP1 were purchased from Abcam (MA, US, ab171739). The statistical analysis was conducted by the GraphPad Prism software 8.0 (GraphPad Software, Inc., San Diego, CA, USA). Data are represented as means ± standard deviations. The expression level of FABP1 in gastric cancer tissues and adjacent tissues was analyzed by Student's *t*-test, the Chi-square test was used for the analysis of clinicopathological features, and the Kaplan–Meier method and the log-rank test were used for survival analysis. *P* < 0.05 was considered statistically significant.

## 3. Results

### 3.1. Differential Gene Expression Analysis

**Figure 2:**
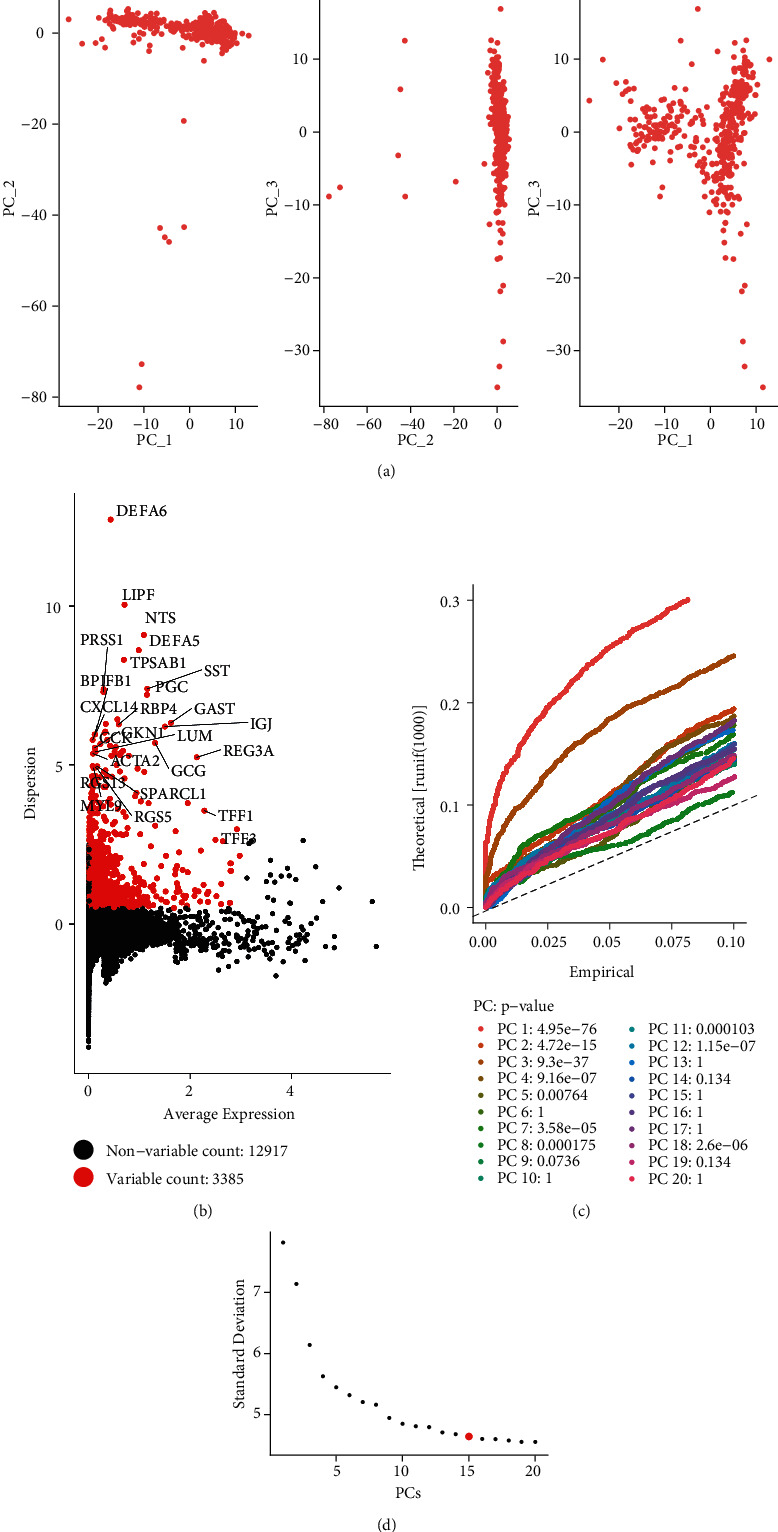
The single-cell transcriptome (scRNA) analysis of early gastric cancer (EGC). (a) The principal component analysis (PCA) results suggested that cells of EGC have consistency in subsequent analysis. (b) The variable feature plot showed the variable regulators in EGC development. (c, d) The Jack-Straw and Elbow plots were applied to select the best cell components in scRNA analysis.

Using the analytical methods described in Methods, we filtered out cells with unique feature counts over 4000 or less than 200. The sum of 3385 variable features expressed in 4110 early gastric cancer (EGC) cells was subjected to scRNA-seq background correction, normalization, and differentially expressed (DE) analysis (Figures 2(a) and 2(b)). A total of 15 PCs were found to simultaneously meet the selection criteria of the contribution degree model and PCA analysis (Figures 2(c) and 2(d)).

### 3.2. tSNE Cell Subtype Detection

**Figure 3:**
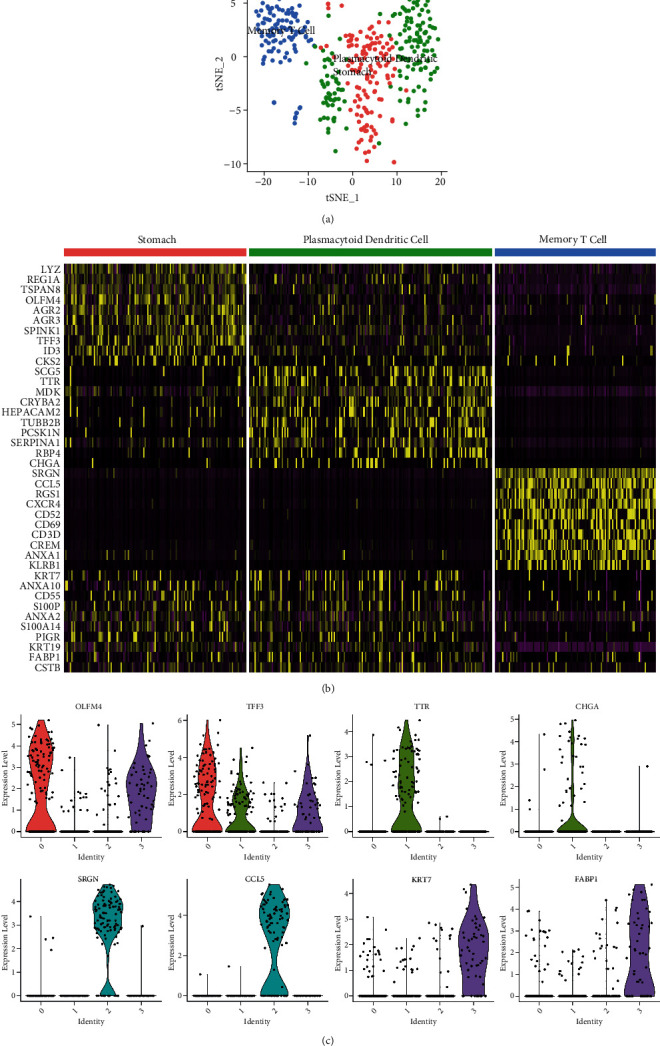
The cell annotation and differentially expressed genes (DEGs) identification. (a) The cell annotation results were presented by tSNE map based on the scCATCH algorithm. (b) The DEGs among the different cell clusters are shown in the heatmap. (c) The expression levels of primarily DEGs in different cell clusters.

After annotation and identification, we detected the gastric cells, plasmacytoid dendritic cells, and memory T cells. The corresponding top DEGs expression profile is presented in Figure 3(b). The expression levels of the top 8 hub genes in the corresponding cell clusters are shown in Figure 3(c).

### 3.3. Biological Function Analysis of Differential Genes

**Figure 4:**
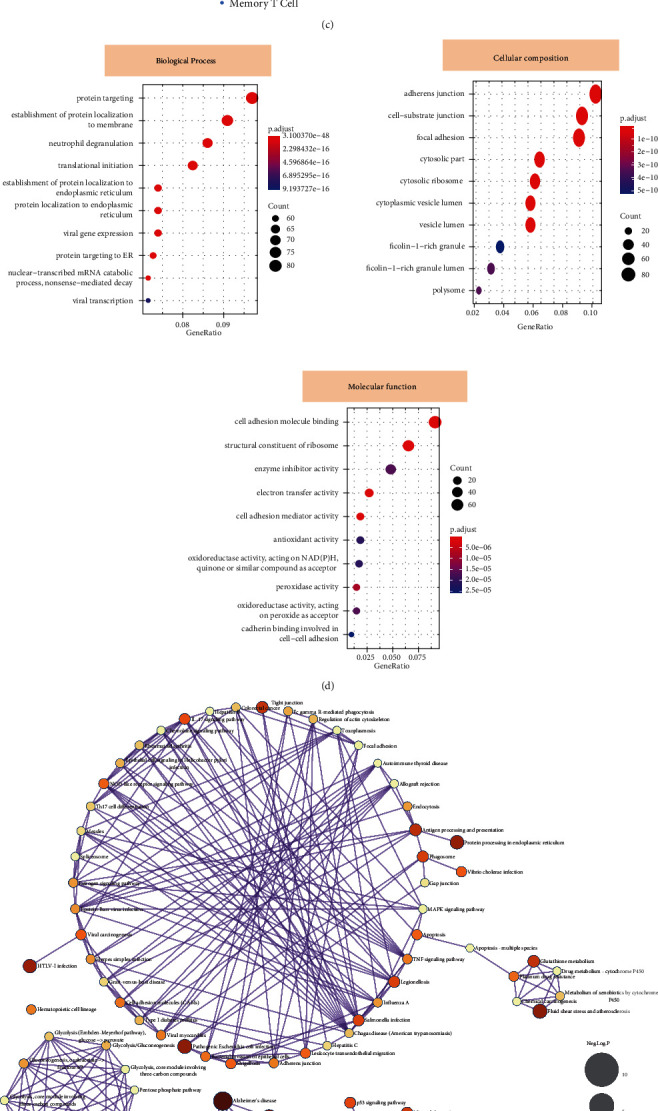
The pseudo-time-related differentially expressed genes (DEGs) detection and enrichment analysis of biological functions. After selecting the appropriate cell principal components (a), the pseudo-time analysis was applied based on the monocle algorithm (b, c). (d) The analysis results presented the GO functional analysis results of biological processes (BP), cellular components (CC), and molecular functions (MF), respectively. (e) The results of the KEGG pathway enrichment analysis. The nodes' size represented the negative log10 *p* value for illustrative purposes. (f) The volcano plot showed the differentially expressed genes in pseudo-time analysis. (g) The compartmentalized protein-protein interaction of FABP1 was constructed based on the COMPPI database.

After selecting the appropriate cellular principal components (Figure 4(a)), we performed a pseudo-time analysis based on the monocle algorithm with the data reduction by the DDtree method (Figures 4(b) and 4(c)). In addition, we further analyzed the most significant GO functions and KEGG pathways of DEGs by the pseudo-time analysis. The significant GO functions mainly involved three main aspects: biological processes (BP), cellular components (CC), and molecular functions (MF). In terms of BP-related functions, the DEGs were mainly associated with protein targeting (enriched genes = 68, *P* value = 3.85E-36), the establishment of protein localization to the membrane (enriched genes = 62, *P* value = 2.78E-26), and neutrophil degranulation (enriched genes = 58, *P* value = 4.01E-22). For CC functions, the DEGs are closely related to an adherent junction (enriched genes = 48, *P* value = 1.05E-10), cell-substrate junction (enriched genes = 43, *P* value = 1.85E-10), and focal adhesion (enriched genes = 38, *P* value = 2.23E-10). In terms of MF, DEGs are mainly associated with cell adhesion molecule binding (enriched genes = 51, *P* value = 5.12E-06), structural constituent of ribosome (enriched genes = 42, *P* value = 4.87E-06), and enzyme inhibitor activity (enriched genes = 28, *P* value = 2.91E-05). Visualization of the GO functions occupied by DEGs is shown in Figure 4(d). In terms of their KEGG pathways, the DEGs were significantly correlated with oxidative phosphorylation (enriched genes = 45, *P* value = 3.21E-12), protein processing in the endoplasmic reticulum (enriched genes = 38, *P* value = 2.75E-10), and TNF signaling pathway (enriched genes = 31, *P* value = 1.85E-09). The KEGG pathway enrichment results are shown in Figure 4(e).

### 3.4. Hub Marker Detection and COMPPI Network Analysis

The volcano map in Figure 4(f) shows the average fold changes in the expression of the marker and adjusted *P* value (the fold change value is mainly based on ComPPI to analyze the interaction regulation of target proteins at the subcellular level to explore the possible regulation or interaction proteins at the levels of the nucleus, cytoplasm, mitochondria, and cell membrane). Using the COMPPI database and Cytoscape software, network diagrams were generated and FABP1 targeted genes in the network were screened based on the differential cellular locations of correlated connectivity. In this part, we targeted the FABP1, and thus, 7 correlated proteins, located in the cytosol, mitochondrion, nucleus, extracellular secretory pathway, and membrane, were intercepted to construct the PPI network in Figure 4(g).

**Figure 5:**
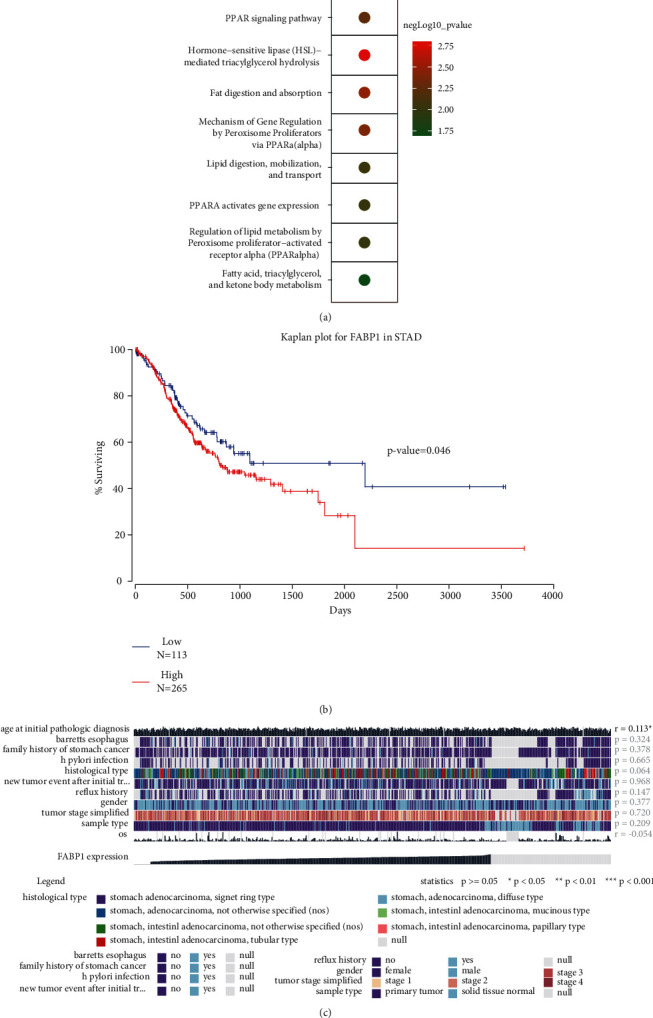
The function enrichment and survival analysis of a hub regulator. (a) The biological process analysis of FABP1 was performed based on the ToppGene database. (b) The overall survival analyses of FABP1 in gene expression values were applied based on TCGA-STAD tissues. (c) The identification of the relationship between the FABP1 and clinical characteristics of patients with gastric cancer in the TCGA-STAD dataset.

In Figure 5(a), results indicated that the biological functions of FABP1 were significantly correlated with hormone-sensitive lipase (HSL)-mediated triacylglycerol hydrolysis (*P* value = 0.0032), fat digestion and absorption (*P* value = 0.0047), and mechanism of gene regulation by peroxisome proliferators via PPAR-alpha (*P* value = 0.0051).

### 3.5. Survival Analysis

Figure 5(b) shows that the expression of FABP1 had a significant influence on the overall survival (OS) of STAD patients (*P* value = 0.046) in the SurvExpress database. Besides, the expression level of FABP1 was also correlated with age at initial pathologic diagnosis (*P* < 0.05 and *R* = 0.31) and OS (*P* < 0.05 and *R* = 0.44) in the Wanderer database (Figure 5(c)).

### 3.6. Upregulation of FABP1 in GC and Its Correlation with Poor Prognosis

**Figure 6:**
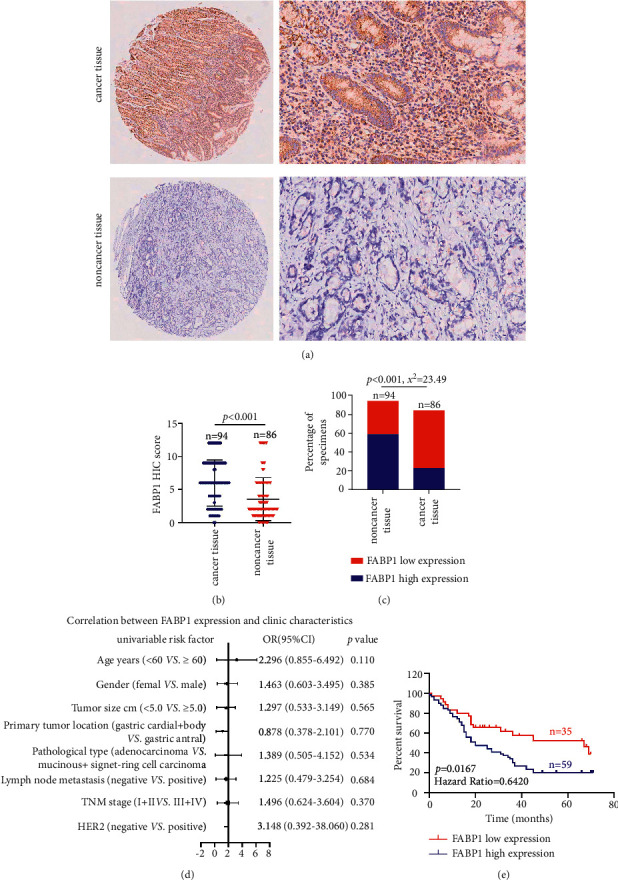
The expression and clinical significance of FABP1 in gastric cancer. (a, b) Tissue microarray (TMA) analysis by IHC staining showed that a high expression of FABP1 was observed in gastric cancer tissues. (c) High and low expression rates of FABP1 in gastric cancer tissue. (d) Correlations of FABP1 expression levels in gastric cancer tissues and clinicopathological features. (e) Kaplan–Meier analysis showed that gastric cancer patients with upregulation of FABP1 were positively correlated with worse prognosis and shorter overall survival.

To further investigate the expression of FABP1 in GC tissues and its relationship with the prognosis of GC patients, we analyzed the expression of FABP1 in GC tissue microarray by IHC. The results showed that the expression level of FABP1 in GC tissues was higher than that in the noncancer tissues (Figures 6(a) and 6(b)), and the high expression rate of FABP1 in gastric cancer tissues was higher than that in noncancer tissues (Figure 6(c)). However, the expression of FABP1 was not significantly correlated with clinical-pathological features such as age, gender, tumor size, histopathological type, lymph node positive, TNM stage, and HER2 positive (Figure 6(d) and Table 1). More importantly, Kaplan–Meier analysis indicated that upregulation of FABP1 was consistently correlated with a worse prognosis (Figure 6(d)), suggesting a tumor promotion role and prognostic value of FABP1 in GC.

## 4. Discussion

Studies have shown that about 70% of GC patients have already developed liver and peritoneal metastasis at their first visit [28]. As a result, improving the early diagnosis rate of GC is critical to promoting the survival rate of GC patients. Most GC patients are failed to accept medical examinations in time due to the atypical symptoms. The popularization and promotion of GC screening methods are particularly important. Currently, the diagnostic methods of GC are mainly endoscopy, imaging examination, and serum markers. However, many patients cannot accept the endoscopy because it is an invasive examination. In addition, the lack of skills and experience of endoscopists and pathologists lead to the early missed diagnosis. It is also difficult to detect small lesions by imaging examination, and the disease may be already in an advanced stage when positive symptoms are detected. Nowadays, the serum markers used for the diagnosis of GC are mainly CEA, CA 19-9, and CA72-4. However, these serum markers are not or less expressed in some GC, leading to false negatives and early misdiagnosis of GC. Therefore, it is important to explore and develop new biomarkers with high sensitivity and specificity for the early diagnosis of GC.

Recently, as an emerging sequencing technology, single-cell RNA sequencing can further explore the heterogeneity of malignant tumors, tumor evolution, clinical diagnosis, and treatment at different omics levels of single cells [29]. The key regulatory factors of various tumor cells have been identified using single-cell RNA sequencing technology, including factors in the immune microenvironment, drug resistance, and metastasis. Some researchers conducted single-cell RNA sequencing analysis of human liver cancer T cells and found that there are many dysfunctional CD8^+^ T cells and regulatory T cells in tumor tissue. By analyzing the DEGs of the two types of cells, they found that the gene Layilin can inhibit the killing function of CD8^+^ T cells and may become a potential target for liver cancer immunotherapy [30]. In esophageal cancer cell lines resistant to paclitaxel, single-cell RNA sequencing results showed that proteasome genes and HIF-1 signaling genes were associated with acquired paclitaxel resistance in esophageal cancer cells [31]. In a colorectal cancer study, single-cell RNA sequencing analysis was performed, respectively, on the primary, metastatic, and circulating tumor cells of the metastatic colorectal cancer. Results showed that circulating tumor cells have not only the same mutated driver genes (such as APC, KRAS, or PIK3CA) with the primary and metastatic lesions but also new variant genes [32].

In this study, we analyzed the single-cell RNA sequencing data of GC. Firstly, we analyzed 3385 variable cell features expressed by 4110 EGC cells. After annotation and identification, we detected gastric cells, plasmacytoid dendritic cells, and memory T cells. Expression levels of the top 8 (OLFM4, TFF3, TTR, CHGA, SRGN, CCL5, KRT7, and FABP1) hub genes of the corresponding cell clusters were also identified. In addition, using the pseudo-time analysis, we further analyzed the most significant GO functions and KEGG pathways of DEGs, revealing the regulatory effects of DEGs on the biological function of GC according to biological processes, cellular components, and molecular functions, as well as the closed relationship between the DEGs and oxidative phosphorylation, endoplasmic reticulum protein processing, and TNF signaling pathways. Moreover, we identified the significant DEG-FABP1 and found that FABP1 may regulate the PPAR signaling pathway, hormone-sensitive lipase (HSL)-mediated triacylglycerol hydrolysis, fat digestion, and absorption in gastric cancer progression. Survival analysis showed that higher FABP1 expression predicts a lower survival rate in GC patients. The expression of FABP1 is also correlated with the age of patients at initial pathological diagnosis. Single-cell RNA sequencing can obtain genomic and transcriptome information of cancer center cells, pericancerous cells, and distant metastasis cancer cells, so as to find effective therapeutic targets for cancer. For our analysis, we found the abnormal expression of FABP1 in early gastric cancer tissues by analyzing the data of single-cell RNA sequencing, which plays a very important role in the treatment of gastric cancer. In recent years, a number of studies have reported the use of single-cell RNA sequencing to find treatment for gastric cancer. Immune cells and stromal cells were found to exhibit cellular heterogeneity in tissues with distant metastases from gastric cancer, and genes regulating CD8+ cell depletion were screened [33]. Both inflammatory cancer-associated fibroblasts and extracellular matrix cancer-associated fibroblasts can mobilize surrounding immune cells to build a microenvironment conducive to the growth of gastric cancer cells [34]. These results obtained by single-cell RNA sequencing undoubtedly can provide new ideas for the treatment of gastric cancer.

FABP1 is a low-molecular-weight protein composed of 127 amino acids. As a lipid chaperone, each FABP1 molecule can bind to two long-chain fatty acid molecules. FABP1 can also bind to other hydrophobic ligands to regulate various biological processes such as cell growth, differentiation, and apoptosis [35]. Studies have shown that FABP1 is detected in about 38% of GC patients, mainly in gastric papillary adenocarcinoma, female cases, and patients with age less than 50. FABP1 is highly expressed in gastric intestinal metaplasia and gastric adenocarcinoma tissues, but not or less expressed in gastric tissues [36]. We performed IHC by GC tissue microarray and found that the expression level of FABP1 was significantly higher in the GC tissues than in the adjacent tissues. Furthermore, GC patients with higher expression of FABP1 had a worse prognosis. Previously, researchers had reported that FABP1 is expressed in early-stage GC, with a specificity of 95% and a sensitivity of 67% for the diagnosis of early recurrence, and patients with multiple positive results of this gene have a worse prognosis [37].

Interestingly, the expression of FABP1 has no significant correlation with clinicopathological features such as age, sex, tumor size, histopathological type, lymph node positivity, TNM stage, and HER2 positivity. However, some researchers reported that FABP1 expression was detected in the peritoneal lavage fluid of GC patients, and the prognosis of FABP1-positive patients was worse than that of CEA. At least half of them had a peritoneal recurrence, and the recurrence rate was 67% [38]. The results reported are inconsistent with ours, which may be caused by the diversity of clinical samples. In our future study, we will expand the sample size to clarify these issues, and the content of FABP1 can be detected in serum and feces of patients with a confirmed diagnosis of GC, to prove that FABP1 can be used as a marker for the diagnosis of gastric cancer. In addition, we need to conduct further research in other aspects, such as observing the biological effect of FABP1 on the GC cells after downregulating and upregulating the expression of FABP1 in vitro and in vivo and elucidating the molecular mechanism of FABP1 promoting cancer from different perspectives in terms of fat metabolism.

In conclusion, we found that FABP1 is a key regulatory gene of GC and is associated with poor prognosis based on the single-cell RNA sequencing data. Tissue microarray analysis also showed that FABP1 is highly expressed in GC tissues, and the survival rate of patients with high FABP1 expression is lower. FABP1 is expected to become a promising marker for early diagnosis and targeted therapy of GC.

## Figures and Tables

**Table 1:** Correlation of the expression of FABP1 in gastric cancer with clinicopathologic features.

Characteristics	No. of patients	FABP1		*P* value
		Low expression, n (%)	High expression, n (%)	
Overall	94	35 (37.2)	59 (62.8)	
Age				0.110
<60 years, n (%)	25 (26.6)	19 (76.0)	6 (24.0)	
≥60 years, n (%)	69 (73.4)	40 (58.0)	29 (42.0)	
Gender				0.385
Female	59 (62.8)	39 (66.1)	20 (33.9)	
Male	35 (37.2)	20 (57.1)	15 (42.9)	
Tumor size				0.565
<5.0 cm	33 (35.1)	22 (66.7)	11 (33.3)	
≥5.0 cm	61 (64.9)	37 (60.7)	24 (39.3)	
Primary tumor location				0.770
Gastric cardial + body	60 (63.8)	37 (61.7)	23 (38.3)	
Gastric antral	34 (36.2)	22 (64.7)	12 (35.3)	
Pathological type				0.534
Adenocarcinoma	78 (83.0)	50 (64.1)	28 (35.9)	
Mucinous + signet-ring cell carcinoma	16 (17.0)	9 (56.3)	7 (43.7)	
Lymph node metastasis				0.684
Negative	72 (76.6)	46 (63.9)	26 (36)	
Positive	22 (23.4)	13 (59.1)	9 (40.9)	
TNM stage				0.370
I + II	35 (37.2)	24 (68.6)	11 (31.4)	
III + IV	59 (62.8)	35 (59.3)	24 (40.7)	
HER2				0.668
Positive	6 (6.4)	5 (83.3)	1 (16.7)	
Negative	88 (93.6)	54 (61.4)	34 (38.6)	

## Data Availability

The data used to support the findings of this study are available from the corresponding authors upon request.

## Abbreviations

FABP1: Fatty acid-binding protein 1
OLFM4: Olfactomedin 4
TFF3: Trefoil factor3
TTR: Transthyretin
CHGA: Chromogranin A
SRGN: Proteoglycan serglycin
CCL5: C-C chemokine ligand 5
KRT7: Keratin 7.

## References

[1] Bray F., Ferlay J., Soerjomataram I., Siegel R. L., Torre L. A., Jemal A. (2018). Global cancer statistics 2018: GLOBOCAN estimates of incidence and mortality worldwide for 36 cancers in 185 countries. *CA: A Cancer Journal for Clinicians*.

[2] Oh S. E., An J. Y., Choi M. G., Lee J. H., Sohn T. S., Bae J. M. (2020). Comparisons of remnant primary, residual, and recurrent gastric cancer and applicability of the 8th AJCC TNM classification for remnant gastric cancer staging. *European Journal of Surgical Oncology*.

[3] Japanese Gastric Cancer A. (2021). Japanese gastric cancer treatment guidelines 2018. *Gastric Cancer*.

[4] Zeng Z., Li W., Zhang D. (2022). Development of a chemoresistant risk scoring model for prechemotherapy osteosarcoma using single-cell sequencing. *Frontiers in Oncology*.

[5] Li L., Xiong F., Wang Y. (2021). What are the applications of single-cell RNA sequencing in cancer research: a systematic review. *Journal of Experimental & Clinical Cancer Research*.

[6] Furuhashi M., Hotamisligil G. S. (2008). Fatty acid-binding proteins: role in metabolic diseases and potential as drug targets. *Nature Reviews Drug Discovery*.

[7] Xu H., Diolintzi A., Storch J. (2019). Fatty acid-binding proteins: functional understanding and diagnostic implications. *Current Opinion in Clinical Nutrition and Metabolic Care*.

[8] Chmurzyńska A. (2006). The multigene family of fatty acid-binding proteins (FABPs): function, structure and polymorphism. *Journal of Applied Genetics*.

[9] Huang H., McIntosh A. L., Martin G. G. (2016). FABP1: a novel hepatic endocannabinoid and cannabinoid binding protein. *Biochemistry*.

[10] Schroeder F., McIntosh A. L., Martin G. G. (2016). Fatty acid binding protein-1 (FABP1) and the human FABP1 T94A variant: roles in the endocannabinoid system and dyslipidemias. *Lipids*.

[11] Wang G., Bonkovsky H. L., de Lemos A., Burczynski F. J. (2015). Recent insights into the biological functions of liver fatty acid binding protein 1. *Journal of Lipid Research*.

[12] Smathers R. L., Petersen D. R. (2011). The human fatty acid-binding protein family: evolutionary divergences and functions. *Human Genomics*.

[13] Srivastava S., Kern F., Sharma N. (2017). FABP1 and Hepar expression levels in Barrett’s esophagus and associated neoplasia in an Asian population. *Digestive and Liver Disease*.

[14] Sharaf R. N., Butte A. J., Montgomery K. D., Pai R., Dudley J. T., Pasricha P. J. (2011). Computational prediction and experimental validation associating FABP-1 and pancreatic adenocarcinoma with diabetes. *BMC Gastroenterology*.

[15] Zhang G. L., Pan L. L., Huang T., Wang J. H. (2019). The transcriptome difference between colorectal tumor and normal tissues revealed by single-cell sequencing. *Journal of Cancer*.

[16] Wood S. M., Gill A. J., Brodsky A. S. (2017). Fatty acid-binding protein 1 is preferentially lost in microsatellite instable colorectal carcinomas and is immune modulated via the interferon *γ* pathway. *Modern Pathology: An Official Journal of the United States and Canadian Academy of Pathology*.

[17] Barrett T., Wilhite S. E., Ledoux P. (2012). NCBI GEO: archive for functional genomics data sets--update. *Nucleic Acids Research*.

[18] Zhang P., Yang M., Zhang Y. (2019). Dissecting the single-cell transcriptome network underlying gastric premalignant lesions and early gastric cancer. *Cell Reports*.

[19] Mangiola S., Doyle M. A., Papenfuss A. T. (2021). Interfacing Seurat with the R tidy universe. *Bioinformatics*.

[20] Satija R., Farrell J. A., Gennert D., Schier A. F., Regev A. (2015). Spatial reconstruction of single-cell gene expression data. *Nature Biotechnology*.

[21] David C. C., Jacobs D. J. (2014). Principal component analysis: a method for determining the essential dynamics of proteins. *Methods in Molecular Biology*.

[22] Shao X., Liao J., Lu X., Xue R., Ai N., Fan X. (2020). scCATCH: automatic annotation on cell types of clusters from single-cell RNA sequencing data. *iScience*.

[23] Zhou Y., Zhou B., Pache L. (2019). Metascape provides a biologist-oriented resource for the analysis of systems-level datasets. *Nature Communications*.

[24] Veres D. V., Gyurkó D. M., Thaler B. (2015). ComPPI: a cellular compartment-specific database for protein-protein interaction network analysis. *Nucleic Acids Research*.

[25] Chen J., Bardes E. E., Aronow B. J., Jegga A. G. (2009). ToppGene Suite for gene list enrichment analysis and candidate gene prioritization. *Nucleic Acids Research*.

[26] Aguirre-Gamboa R., Gomez-Rueda H., Martínez-Ledesma E. (2013). SurvExpress: an online biomarker validation tool and database for cancer gene expression data using survival analysis. *PLoS One*.

[27] Deng H. M., Huang L., Liao Z. K., Liu M., Li Q., Xu R. (2020). Itraconazole inhibits the Hedgehog signaling pathway thereby inducing autophagy-mediated apoptosis of colon cancer cells. *Cell Death & Disease*.

[28] Riihimäki M., Hemminki A., Sundquist K., Sundquist J., Hemminki K. (2016). Metastatic spread in patients with gastric cancer. *Oncotarget*.

[29] Suva M. L., Tirosh I. (2019). Single- cell RNA sequencing in cancer: lessons learned and emerging challenges. *Molecular Cell*.

[30] Zheng C., Zheng L., Yoo J. K. (2017). Landscape of infiltrating T cells in liver cancer revealed by single-cell sequencing. *Cell*.

[31] Wu H., Chen S., Yu J. (2018). Single-cell transcriptome analyses reveal molecular signals to intrinsic and acquired paclitaxel resistance in esophageal squamous cancer cells. *Cancer Letters*.

[32] Heitzer E., Auer M., Gasch C. (2013). Complex tumor genomes inferred from single circulating tumor cells by array-CGH and next-generation sequencing. *Cancer Research*.

[33] Jiang H., Yu D., Yang P. (2022). Revealing the transcriptional heterogeneity of organ-specific metastasis in human gastric cancer using single-cell RNA Sequencing. *Clinical and Translational Medicine*.

[34] Li X., Sun Z., Peng G. (2022). Single-cell RNA sequencing reveals a pro-invasive cancer-associated fibroblast subgroup associated with poor clinical outcomes in patients with gastric cancer. *Theranostics*.

[35] Prinetti A., Mitro N. (2016). FABP1 in wonderland. *Journal of Neurochemistry*.

[36] Hashimoto T., Kusakabe T., Watanabe K. (2004). Liver-type fatty acid-binding protein is highly expressed in intestinal metaplasia and in a subset of carcinomas of the stomach without association with the fatty acid synthase status in the carcinoma. *Pathobiology*.

[37] Jiang Z., Shen H., Tang B. (2017). Identification of diagnostic markers involved in the pathogenesis of gastric cancer through iTRAQ-based quantitative proteomics. *Data in Brief*.

[38] Kodera Y., Nakanishi H., Ito S. (2002). Quantitative detection of disseminated free cancer cells in peritoneal washes with real-time reverse transcriptase-polymerase chain reaction: a sensitive predictor of outcome for patients with gastric carcinoma. *Annals of Surgery*.

